# The pharmaceuticalisation of security: Molecular biomedicine, antiviral stockpiles, and global health security

**DOI:** 10.1017/S0260210514000151

**Published:** 2014-12

**Authors:** STEFAN ELBE

## Abstract

Pharmaceuticals are now critical to the security of populations. Antivirals, antibiotics, next-generation vaccines, and antitoxins are just some of the new ‘medical countermeasures’ that governments are stockpiling in order to defend their populations against the threat of pandemics and bioterrorism. How has security policy come to be so deeply imbricated with pharmaceutical logics and solutions? This article captures, maps, and analyses the ‘pharmaceuticalisation’ of security. Through an in-depth analysis of the prominent antiviral medication *Tamiflu*, it shows that this pharmaceutical turn in security policy is intimately bound up with the rise of a molecular vision of life promulgated by the biomedical sciences. Caught in the crosshairs of powerful commercial, political, and regulatory pressures, governments are embracing a molecular biomedicine promising to secure populations pharmaceutically in the twenty-first century. If that is true, then the established disciplinary view of health as a predominantly secondary matter of ‘low’ international politics is mistaken. On the contrary, the social forces of health and biomedicine are powerful enough to influence the core practices of international politics – even those of security. For a discipline long accustomed to studying macrolevel processes and systemic structures, it is in the end also our knowledge of the minute morass of molecules that shapes international relations.

## Introduction

The rapid rise of global health security as a new policy domain testifies to the increased attention that health issues are receiving in international politics. A seeming epidemic of epidemic threats (from HIV/AIDS, SARS, H5N1, H1N1, through to MERS, H7N9, and Ebola) has forced governments to grapple with the question of how to best protect their populations and economies against acute infectious disease threats.^1^ Defined officially by the World Health Organization (WHO), global health security encompasses ‘the activities required, both proactive and reactive, to minimize vulnerability to acute public health events that endanger the collective health of populations living across geographical regions and international boundaries’.^2^ Such policies are necessary, according to WHO, because a new pandemic infecting roughly 25 per cent of the world population (a figure derived from previous pandemics), would affect more than 1.5 billion people and cause enormous social disruption due to a rapid surge in illnesses and deaths.^3^ The threat of bioterrorism – exemplified by the anthrax letters mailed in the United States in the autumn of 2001 – similarly demands ongoing government efforts to prepare for the deliberate release of a biological agent. The twin spectres of naturally-occurring and intentionally-released infectious disease threats have thus provoked a deep sense of microbial unease at the outset of the twenty-first century.

Security agendas have evolved to reflect this mood shift, and now routinely incorporate health-based threats. In the United States, the 2002 National Security Strategy made direct reference to infectious diseases, pledging that the US government will ‘continue to lead the world in efforts to reduce the terrible toll of HIV/AIDS and other infectious diseases’.^4^ The 2006 US National Security Strategy again pointed to the threat posed by ‘public health challenges like pandemics (HIV/AIDS, avian influenza) that recognise no borders’.^5^ When the United Kingdom developed its first official national security strategy in 2008, it too began to highlight pandemic threats – both because of their ability to affect the country, and because they could potentially undermine international stability.^6^ Pandemic threats also continue to reside at the apex of the UK's national risk register, and are identified as a Tier 1 (top) threat in the latest National Security Strategy.^7^ Those UK efforts, in turn, unfolded against the backdrop of wider European Union initiatives to also develop a European health security strategy.^8^ In a way that would have been unimaginable only a decade ago, potentially catastrophic infectious disease threats have become the unlikely bedfellows of more established security threats like terrorism, nuclear proliferation, rogue states, and so forth.^9^

The emergence of those global health security concerns creates new challenges and opportunities for International Relations scholarship. First, global health security marks another significant expansion of the international security agenda. At a time when a number of infectious diseases have become the subject of high-level, sustained and even acrimonious international diplomacy, scholars of International Relations are challenged to rectify their neglect of the international politics of health. Second, global health security also opens up new opportunities for investigating the intricate dynamics of securitisation in world politics.^10^ Amidst the pantheon of recent securitisation processes, global health security stands out because it begins to transform the inner biological workings of our bodies into additional sites of security concern.^11^ Here the rise of global health security is engendering a range of new practices that can be fruitfully explored with a view to deepening our understanding of contemporary securitisation processes. For those two reasons alone, a burgeoning International Relations literature has already turned its analytical sites on global health security.^12^

Yet there is also a third – and altogether different – reason why the rise of global health security marks such a significant development in world politics. All the high-level concern around global health security dramatically elevates the role that pharmaceuticals now play in security policy. Acknowledging the dangers of health-based threats has gone hand-in-hand with a heighted government interest in developing new pharmaceutical defences against those very threats. The more governments worry about the threat posed by infectious diseases, the more they come to realise that the security of their populations necessitates things other than organised armed forces; it also requires the ability to implement a range of public health interventions (like travel restrictions and social distancing measures), and ultimately the procurement of large quantities of new pharmaceutical and medical products that can be quickly dispensed to the population in the event that a biological threat should materialise. Strengthening health security over the past decade has thus entailed extensive government investment in the development, acquisition, stockpiling, and distribution of new ‘medical countermeasures’ – like antivirals, antibiotics, next-generation vaccines, and antitoxins.

This article captures, maps, and analyses that pharmaceutical turn in security policy. It argues that the ‘pharmaceuticalisation’ of security is intimately bound up with the rise of a molecular vision of life promulgated by the biomedical sciences. As the article shows through the first in-depth case study of the prominent antiviral medication *Tamiflu*, that molecular knowledge forms the scientific basis for the pharmacological development of many new medical countermeasures. Once created, the commercial backers of such innovative molecular therapies then seek to maximise their financial return on investment by trying to persuade governments to procure large quantities of their new products. At the same time, citizens themselves also increasingly expect their governments to facilitate rapid access to the latest pharmaceutical treatments – especially during a national emergency. Caught in the crosshairs of those combined commercial and political pressures, governments are adapting their regulatory regimes to make innovative medical countermeasures more readily and widely accessible.

All of this implies that the conventional disciplinary view of health as a predominantly secondary matter of ‘low’ international politics is incorrect. On the contrary, this article points to social forces of health and biomedicine that are sufficiently powerful to shape the core practices of international politics – even those of security. Looking more closely at the role of pharmaceuticals in security policy reveals that, far from being tangential to international studies, health and medicine in fact play a constitutive role in our understandings, analyses, and practices of international relations.^13^

## I. Medical countermeasures: Securing populations pharmaceutically

If there is one concept that succinctly captures the pharmaceutical turn in security policy it is this: ‘medical countermeasures’. The exact definition of the term, which has recently gained extensive policy traction, is far from settled. One attempt to define it emanates from the influential Institute of Medicine (IOM) in the United States: ‘a drug, biological product, or device that treats, identifies, or prevents harm from a biological, chemical, radiological, or nuclear agent that may cause a public health emergency’.^14^ Technicalities of the definition aside, the concept of medical countermeasures broadly refers to pharmaceutical interventions that can be mass administered to populations in the event of a deliberate or naturally occurring security threat materialising.

It is a treasure trove of a concept in that it manages – in a single idea – to combine at least three fascinating and intertwined developments. On the one hand, the term expresses the keen interest that governments currently display in developing novel pharmaceutical defences against security threats. On the other hand, the term also textually embodies the progressive epistemic blending of the professional worlds of medicine (‘medical’) and security/defence (‘countermeasures’) – neatly merging key vocabularies from both expert communities. Finally, the term reflects the hardening aspiration of governments to extend such pharmaceutical protections beyond the battlefield, and to cover much larger civilian populations. Consider, for example, the Public Health Emergency Medical Countermeasure Enterprise (PHEMCE) in the United States. Citing the need to defend American *citizens* against health security threats, it takes the lead in ‘protecting the civilian population from potential adverse health impacts through the use of medical countermeasures, which are medicines, devices, or other medical interventions that can lessen the harmful effects of these threats’.^15^ In a single notion, the concept of medical countermeasures captures how security policy has recently gravitated much more strongly towards pharmaceutical solutions, how the concerns of health and security professionals increasingly interpenetrate each other, and how security planners are broadening out those pharmaceutical protections beyond the military to cover entire populations. It is, in that regard, the quintessential concept of health security.

All of that is no doubt making a lot of demands of a singular concept. Moreover, it is comparatively easy to coin new terms. So, to what extent has the new concept of medical countermeasures also been backed by more meaningful financial commitments? In fact, some governments have already made quite substantial investments in the development of new medical countermeasures. The United States, for example, has expended significant public funds through the BioShield program launched in 2004. The programme sought to accelerate the research, development, purchase, and availability of effective medical countermeasures by establishing a secure source of public funding worth US $5.6 billion. The idea behind that legislation was for the US government to make a credible budgetary commitment to bulk-buy new medical countermeasures, and that this would serve as a financial incentive to lure pharmaceutical companies into the medical countermeasures market.^16^ It is one of the strongest pieces of evidence that governments are doing more than just paying lip service to the quest for novel pharmaceutical defences.

Perhaps, then, all of this political activity around medical countermeasures is simply a knee-jerk political reaction to the extraordinary events of 11 September 2001, and the subsequent mailing of the anthrax letters in the United States? Not so. For the US government has also demonstrated its longer-term commitment to this area through the creation of a whole new institution tasked with realising the new medical countermeasures mission – the Biomedical Advanced Research and Development Authority (BARDA) established in 2006. The organisation's primary strategic goal is to create an ‘advanced development pipeline replete with medical countermeasures and platforms to address unmet public health needs, emphasizing innovation, flexibility, multi-purpose and broad spectrum application, and long-term sustainability’.^17^ Further goals of the organisation include the maintenance of an ‘agile, robust and sustainable U.S. manufacturing infrastructure capable of rapidly producing vaccines and other biologics against pandemic influenza and other emerging threats’.^18^ Over a decade after the pivotal events of 2001, the US government's political commitment to the medical countermeasure enterprise continues, as evidenced by its recent decision to reauthorise BARDA for another five years through passing the Pandemic All Hazards Preparedness Reauthorisation Act of 2013.

Since its inception, BARDA has initiated and/or completed acquisition contracts for new medical countermeasures worth more than US $2 billion – on anthrax antitoxins and vaccines, botulism therapeutics, smallpox vaccine, and radiological, nuclear, and chemical threats.^19^ The extent to which Project Bioshield has been successful remains a topic of considerable debate. However, the US government has already been able to add 11 new products to the nation's emergency stockpile under the programme; and – according to the Assistant Secretary for Preparedness and Response, Nicole Lurie – there are a further eighty pharmaceuticals in various stages of development.^20^ Those contracts have led to federal acquisitions totalling tens of millions of doses of medical countermeasures.^21^ The acquisitions come, furthermore, against the background of a wider US government investment of around US $79 billion in civilian biodefense research made since 2001, and in addition to any classified research conducted in the security and defence agencies.^22^

Evidence abound, then, that pharmaceuticals have become much more pivotal to security policy. A new conceptual vocabulary has been forged in the form of ‘medical countermeasures’ – allowing pharmaceutical responses to be closely integrated into security policy. Substantial public treasure has been earmarked and allocated for the commercial development of new pharmaceutical defences. And entire new institutions have been created with the explicit mission of working more closely with pharmaceutical companies to develop and procure innovative medical countermeasures. At the outset of the twenty-first century, security policy is becoming more deeply infused with pharmaceutical logics and rationalities.

## II. Pharmaceutical stockpiling: The logistics of global health security

The pharmaceutical turn in security policy entails more than just the scientific development of novel medical countermeasures. Securing populations pharmaceutically also demands that governments are able to deliver the right drugs, to the right people, at the right time. Here public officials have had to undertake further and extensive logistical planning, culminating in the instigation of another novel security practice: pharmaceutical stockpiling. There is, to be sure, nothing new about stockpiling *per se*; in fact, historians can trace the practice back for thousands of years. Even during the latter half of the twentieth century, many strategic supplies – including hospital and medical supplies – were stockpiled in the context of the Cold War.

Efforts to strengthen health security, however, have given rise a new type of stockpile – one dedicated predominantly, and even exclusively, to pharmaceuticals. In 1999, and against the background of growing concerns about bioterrorism and natural disasters, the United States Congress tasked the Clinton administration with creating a new National Pharmaceutical Stockpile (NPS) that would supply states and communities with large quantities of essential medical material within 12 hours of a government decision.^23^ The new stockpile was limited in size, originally supported with a comparatively modest allocation of US $51 million. Even more significant than its initial size, however, is the fact that this new stockpile was now dedicated specifically to pharmaceuticals.

In any case, the attacks of 11 September 2001 and the subsequent anthrax letters would soon alter the picture dramatically. In 2003, the stockpile was renamed the Strategic National Stockpile (SNS), as it rapidly evolved into a much wider ‘national repository of antibiotics, chemical antidotes, antitoxins, life-support medications, IV administration and airway maintenance supplies, and medical/surgical items’.^24^ Those stockpiled medical countermeasures are now stored in a large number of pre-packed ‘push’ pallets, so that they can be delivered anywhere in the United States at short notice. By 2006, such SNS packages reportedly filled 124 cargo containers, weighing 94,424 pounds and taking up 5,000 square feet of floor space.^25^ Two years later, by 2008, the total inventory of the stockpile was valued at US $3.5 billion.^26^

The precise geographic location and detailed composition of this stockpile remains classified in order to prevent a run on the supplies during an emergency. However, the US government has disclosed that the stockpile was first deployed on 11 September 2001. Of the four airplanes reportedly cleared to fly in American airspace that night, one was Air Force One – with the remaining three supporting the SNS deployment.^27^ The decision to create, and deploy, such dedicated pharmaceutical stockpiles shows that governments are doing more than simply investing in the development of novel medical countermeasures; they are also adapting their security practices to deliver those pharmaceuticals to the population much more rapidly during an emergency.

Perhaps, then, this move towards pharmaceutical stockpiling in security policy is just a peculiarly American phenomenon? The United States, after all, is home to the world's largest pharmaceutical market. It is also one of the few countries in the world where pharmaceuticals can be directly advertised to consumers. And it is a country where the pharmaceutical lobby yields substantial political influence.

There is no doubt that the United States has been at the international forefront of pharmaceutical stockpiling for security purposes; but it would be erroneous to simply dismiss the rise of pharmaceutical stockpiling as a US phenomenon. In fact, the practice of pharmaceutical stockpiling has also been adopted by many other countries around the world. More recently, the European Union established a new legal basis for the voluntary joint procurement of medical countermeasures by member states, especially for influenza vaccines.^28^ The Australian government also created a National Medical Stockpile (NMS) with a strategic reserve of essential vaccines, antibiotics, antiviral drugs, as well as chemical and radiological antidotes.^29^ The Canadian government similarly maintains a National Emergency Stockpile System,^30^ whilst the United Kingdom too has assembled a Reserve National Stock for Major Incidents including nerve agent antidotes, antitoxins, antibiotics, and other post-exposure medications – albeit on a smaller scale than in the United States.^31^

In other areas of global health security, furthermore, the United Kingdom's pharmaceutical stockpiling efforts trump even those of the United States government. As part of its pandemic preparedness planning, UK authorities created one of the world's largest stockpiles of antiviral medications in 2005. Amidst fears that H5N1 (‘bird’) flu could mutate into a form that would cause a devastating human pandemic, the UK government identified the antiviral medication oseltamivir (brand name: *Tamiflu*) as the ‘first line of defence’. The UK subsequently expended considerable public resources to create a new national stockpile of the drug sufficiently large to cover half of its population. Later, the UK government increased the size of this antiviral stockpile further still, this time to cover 80 per cent of the population – a far higher percentage than the equivalent US ambition to achieve 25 per cent coverage of its population.

Many other governments in Europe and around the world have been building similar antiviral stockpiles. A review of European pandemic plans published in 2006, found that twenty European countries had already developed an antiviral-drug strategy – a trend that would intensify through 2007.^32^ In a context where, by 2009, a total of 95 governments around the world had reportedly purchased or ordered antiviral stockpiles, pharmaceutical stockpiling cannot be dismissed as a peculiarly US phenomenon.^33^

Even with the creation of all these new pharmaceutical stockpiles, however, a crucial gap in coverage remained. What if a new infectious disease broke out in a low-income country without a stockpile? Would those countries simply be abandoned to endure their tragic and likely horrible epidemiological fate? Amidst fears in 2005 of an imminent H5N1 (‘bird flu’) pandemic, the commercial manufacturer and distributor of *Tamiflu* – Roche – reportedly shared its order book with the head of Influenza Unit at the World Health Organization. Alarmingly, the book revealed that countries from Southeast Asia had placed only very few antiviral orders, even though this was the place that many experts predicted to be the geographical source of a new pandemic. Everyone understood the implications: if an outbreak occurred there, many of those countries would not have access to antivirals.^34^ That realisation not only exposed worrying international inequalities between high- and low-income countries regarding access to medical countermeasures; it also directly threatened the interests of high income countries – especially if the virus were to quickly spread to their populations on the back of an increasingly globalised air transport infrastructure.

Interest therefore quickly turned towards setting up a supplementary *international* antiviral stockpile for rapid deployment to anywhere in the world, and with the hope of stopping a new influenza virus at source. An influential modelling study published in the scientific journal *Nature* in September 2005 showed that such an international stockpile approach could be viable. The paper concluded: ‘elimination of a nascent pandemic may be feasible using a combination of geographically targeted prophylaxis and social distancing measures’, and predicted that ‘a stockpile of 3 million courses of antiviral drugs should be sufficient for elimination’.^35^ By that time WHO, which has played a key role in the rise of pharmaceutical stockpiling, was already in the process of negotiating the donation of three million treatment courses from Roche for the creation of a new international stockpile. In January 2006, this international stockpile was increased by a further two million donated courses earmarked for developing countries.^36^ The stockpile was physically co-located in the United States and Switzerland – from where it could be quickly flown to a major airport anywhere in the world.^37^

It was a risky strategy. Could a pandemic really be stopped in its tracks? What if some of the assumptions in the theoretical modelling were mistaken, or if the virus did not behave according to those assumptions? Moreover, the size of the stockpile was clearly still very modest, especially when compared to the billions of people living in low-income countries. Again, however, what is more significant than the overall size of the stockpile, is the fact that this new international stockpile now extended the geographic ‘blanket’ of antiviral protection to also cover those countries unable to afford their own pharmaceutical supplies. With the creation of the international WHO stockpile, pharmaceutical stockpiling effectively became a global phenomenon.

And still a crucial gap in global health security remained. As public health planners were quick to point out, the mere procurement of new pharmaceutical stockpiles alone would not guarantee security in the event of an outbreak. In fact, those stockpiles would be fairly useless if they were not accompanied by efficient mechanisms for rapidly distributing those medicines to large numbers of people. Relevant government departments would therefore also need to urgently develop new systems of mass pharmaceutical administration. One of the most innovative and prominent examples of such a new logistical system was the launch of the National Pandemic Flu Service (NPFS) in the United Kingdom during the 2009 influenza A (H1N1) ‘swine flu’ pandemic. Faced with an unexpected surge in human H1N1 infections, which was by that time also beginning to place a heavy burden on the National Health Service (NHS), the authorities in England decided to launch a new telephone and internet-based pharmaceutical distribution system that could deliver the antiviral medications directly to members of the population. It was, in the words of one report, the ‘first mass application of non-clinical based triage’.^38^

Once the new pharmaceutical distribution system went live, and after sorting out some of the initial teething issues caused by overwhelming volumes of Internet traffic, obtaining *Tamiflu* became quite straightforward for citizens. It was simply a matter of picking up the phone or going online, connecting to the new website, and ticking a few boxes related to a set of common flu symptoms. If the symptoms criteria were met, citizens were asked to note down a unique reference number to obtain *Tamiflu* from the nearest official collection point – preferably through the use of what British authorities affectionately referred to as their ‘flu buddies’. Not surprisingly, the system was easily open to abuse from those who wanted to create personal stockpiles of the drug. As one manager of a general medical practice noted with exasperation at the time: ‘at present, it might as well be given out on street corners’.^39^ Overall the service reportedly performed 2,732,000 assessments, of which around 1,800,000 resulted in antiviral authorisation.^40^

Although the British antiviral distribution system was one of the world's most wide-ranging and ambitious, all countries investing in pharmaceutical stockpiles needed to develop plans for rolling out medical countermeasures to their populations. Different models considered and/or adopted by governments included the use of national postal systems, relying on commercial logistics companies, on school buses, or even asking the military to undertake that task. In the end, it is really not so significant which model governments selected. The very fact that they engaged in such extensive logistical planning – on top of their attempts to actively stimulate the development of new medical countermeasures – indicates just how pivotal pharmaceuticals have become to security policy. What is arguably a country's highest political priority – ensuring national security – is now closely dependent upon a government's ability not just to actively develop and acquire, but also to stockpile and rapidly disseminate, large volumes of medical countermeasures.

All those developments render global health security more than just another episode of ‘securitisation’ in contemporary world politics. The rise of global health security is also significant because it marks a critical expansion and intensification of the play of pharmaceutical logics in society. Here, in other words, the rise of global health security begins to emerge as part of a much wider social trend towards increased pharmaceuticalisation that can also be witnessed in many other sectors of society. A number of social scientists working in different disciplines (especially Sociology and Anthropology) are already detecting a very similarly proliferation of pharmaceutical rationalities across quite a diverse array of societal domains and locals.^41^ Building upon that rapidly evolving social science literature, we can think of the process through which security policy too is becoming more deeply imbued with pharmaceutical reason as the *‘pharmaceuticalisation’* of security.

## III. Molecular life: *Tamiflu*, antivirals, and biomedicine

This pharmaceutical turn in security policy described above is intimately bound up with the ascendancy of a molecular vision of life promulgated by the biomedical sciences. The sociologist Nikolas Rose argues that the new molecular vision of existence can be usefully contrasted with an older ‘molar’ model of life and medicine revolving around the visible human body – with its limbs, organs, tissues, blood, and so forth. That older ‘clinical gaze’, famously described by Michel Foucault in *The Birth of Clinic*, is now being supplemented and perhaps even supplanted by a new molecular biomedicine that understands life as a ‘set of intelligible vital mechanisms among molecular entities that can be identified, isolated, manipulated, mobilized, recombined, in new practices of intervention, which are no longer constrained by the apparent normativity of a natural vital order’.^42^ This molecular vision of life is closely – and in fact doubly – implicated in the pharmaceuticalisation of security.

First, the reimagination of life as constituted by the complex interplay of molecular structures and processes is stimulating an array of profound new anxieties about the microbiological vulnerabilities underlying our existence. That is certainly true in the case of bioterrorism, where the growing ability to purposefully manipulate life at the molecular level gives rise to new fears about how microorganisms could be deliberately reengineered or synthesised to cause immense harm to populations.^43^ Similarly, our detailed scientific knowledge of the molecular processes unfolding in nature makes us realise that viruses and bacteria are continuously mutating with the potential to give rise to threatening new pandemics in future. As Angus Nicoll, head of the influenza programme at the European Centre for Disease Prevention and Control (ECDC) puts it: ‘policy-makers and politicians are put in a hard place by the prospect of modern influenza pandemics. They don't know when one is going to happen, where it will start or what it will be like. The only certainty is that future influenza pandemics will occur and they will be unpredictable.’^44^ Here, the molecular vision of life is triggering new microbial anxieties.

Yet if the molecular vision of life is inducing an array of new biological insecurities, it is similarly engendering the development of many new molecular technologies for protecting us from those very threats. How we understand life, in other words, is also reflected in the strategies we develop to intervene upon it medically. Nothing, perhaps, exemplifies this better than the antiviral medication *Tamiflu*. We have already seen that governments around the world invested heavily in stockpiling *Tamiflu* for both the treatment and prevention of pandemic influenza. That quickly turned *Tamiflu* it into one of – if not *the* – most prominent medical countermeasure of the past decade. Roche reports that it supplied around 350 million treatment courses (3.5 billion doses) of *Tamiflu* to governments worldwide between 2004 and 2009 alone,^45^ and that more than 50 million people have taken *Tamiflu* around the world.^46^

So what exactly is *Tamiflu*? As an antiviral medication, *Tamiflu* works quite differently from a traditional vaccine. It is comprised of an artificially synthesised molecule that has been deliberately designed to interfere with the processes of viral replication unfolding inside the human body. The pharmaceutical development of *Tamiflu* occurred against the backdrop of great twentieth-century strides achieved in our scientific understanding of the molecular processes surrounding influenza virus replication – especially following successful isolation of the first human influenza virus in 1933. Scientists now know that viruses, including influenza viruses, cannot replicate on their own; to do that they need to insert themselves into existing cells, and then use the cell to make more copies of themselves. The new virus particles subsequently leave the cell again, destroying the host cell in the process, before going on to infect further cells – repeating this cycle again and again.

Yet the accumulated knowledge about those intricate molecular processes involved in viral replication also exposed a crucial ‘catch’ in the process. As the viruses leave the host cell, they become attached to a coating of (sialic) acid found on the surface of the host cell. To leave the cell and infect neighbouring ones, viruses first require the work of a crucial enzyme called neuraminidase – which helps to dissolve this ‘sticky’ acid and free the viruses.^47^ Metaphorically, one can think of neuraminidase as the ‘scissors’ that cut newly formed virus particles free from their host cell. This neuraminidase enzyme is widely identified by the ‘N’ designation in the international virus classifications frequently reported in the media (for example, H5N1, H1N1, H7N9, etc.).

What would happen to influenza viruses in the absence of this enzyme? Without the proper functioning of the neuraminidase, new virus particles would remain ‘stuck’ on the surface of the host cell with nowhere to go, and would therefore not be able to circulate and penetrate other cells – as would be necessary for causing a wider bodily infection. If there could be a way to selectively disrupt, or inhibit, the workings of this crucial neuraminidase enzyme, it could thus mark an entry point for a new type of antiviral medication. *Tamiflu* – and a closely related predecessor drug named *Relenza* – are two attempts to intelligently and artificially design new molecules that would do precisely that. Together they therefore form part of a new class of antiviral therapies called neuraminidase inhibitors.

Yet the deliberate and rational design of such novel molecular therapies – principally by organic chemists – only became possible *after* Australian researchers had first decoded the molecular structure of neuraminidase. Three key developments facilitated that crucial molecular decoding: (1) the deepening of knowledge about the detailed molecular processes involved in virus replication; (2) the emergence of new scientific technologies like x-ray crystallography capable of unravelling complex molecular structures; and (3) advances in computer modelling and chemical pharmacology used for the rational design and synthesis of new molecules. Armed with these new knowledges and technologies, scientists were eventually able to deliberately design an ‘artificial’ new molecule that could bind to a key site in the neuraminidase enzyme, and that could carry out precisely this desired function of inhibiting the enzyme's key role in the process of viral replication. And so one of the world's most prominent medical countermeasures was born. More than any other medical countermeasure, perhaps, the case of *Tamiflu* shows how the molecular vision of life is not just inducing an array of new insecurities; it is also enabling the scientific development of innovative pharmaceutical interventions designed at the level of the molecular. As a necessary epistemic precondition for the technical and material creation of such novel medical countermeasures, the molecular vision of life promulgated by the life sciences lies at the heart of the pharmaceuticalisation of security.

## IV. Biocapital: Business interests and pharmaceutical companies

If molecular knowledge is a necessary precondition for the pharmaceutical turn in security policy, it is not a sufficient one. For the generation of such biomedical knowledge, and its translation into new pharmaceutical treatments, is a capital-intensive activity. Developing new medical countermeasures requires financial investments frequently running into the tens and even hundreds of millions of dollars. Understanding the ways in which capital coalesces around those new molecular knowledges for the purposes of commercial exploitation is therefore another significant driver of the pharmaceuticalisation of security policy.

Kaushik Rajan points to the emergence of biocapital in the 1990s as ‘a particular form of capitalism made specific because of emergent technologies and epistemologies of the life sciences’.^48^ Such biocapital has been decisive for the development of new medical countermeasures like *Tamiflu* in at least two ways. First, speculative venture capital frequently plays a pivotal role in the early stages of drug discovery. The new molecule that would eventually become *Tamiflu* was initially developed at Gilead Sciences in the mid-1990s. Gilead has since risen to become one of the world's most successful biotechnology companies. At the time it was developing *Tamiflu*, however, Gilead was still only a small start-up company in Silicon Valley, California. During that time it was kept afloat with millions of dollars in private venture capital. Without the willingness of venture capitalists to take considerable financial risks on a new biotechnology business that – at the time – was not yet operating at a profit, and which still had an unproven business model, *Tamiflu* is unlikely to have ever been commercially developed. There is some evidence that the volume of such venture biocapital has diminished significantly in recent years, especially following the financial and banking crisis. Yet many of the medical countermeasures developed over the past decade drew upon such venture capital during an early stage of their development cycle.^49^

Once Gilead Sciences had completed the ‘upstream’ work of designing and synthesising the new therapeutic molecule, it then licenced the compound to a more established pharmaceutical company – Roche – in order to undertake the ‘downstream’ work of conducting clinical trials, obtaining regulatory approval, and marketing the new product. Those are some of the most capital-intensive tasks in the drug development cycle, and are easily beyond the reach of most start-up businesses. For that reason the kind of licensing arrangement used for *Tamiflu* has since become very common in the industry. Yet no sooner had the new therapeutic molecule passed downstream into the hands of the multinational pharmaceutical industry, biocapital began to play an additional role – this time through the pursuit of corporate commercial interests.

Investigative journalists have exposed how – in the case of neuraminidase inhibitors for influenza – pharmaceutical companies like Roche were able to rapidly raise awareness about their new drugs amongst international health organisations – even before the companies had secured regulatory approval.^50^ That was achieved by working closely with scientists, who are often also invited by international health organisations to share their expertise. For example, in 1999 – the same year in which Roche was seeking regulatory approval for *Tamiflu* in the United States – the World Health Organization began to raise concern about pandemic influenza and drafted a document entitled *Influenza Pandemic Plan: The Role of WHO and Guidelines for National and Regional Planning.*^51^ The controversial document, which bears the WHO logo and has been repeatedly cited by the industry, warns of serious consequences of a pandemic and highlights the importance of antiviral medications. Referring mostly to the older generation of antivirals – amantadine and rimantadine – the report also pointed to the development of two new compounds – zanamivir and oseltamivir – that would subsequently become marketed as *Relenza* and *Tamiflu* respectively. The report noted: ‘if approved, and found to have a good safety profile, either drug would offer the advantage, during inter-pandemic situations, of being useful regardless of the virus type’.^52^

A subsequent investigation published in *BMJ* (formerly the *British Medical Journal*) further revealed that the document was compiled by WHO in collaboration with the European Scientific Working Group on Influenza (ESWI) – which was industry-funded by Roche and other influenza drug manufacturers.^53^ The *BMJ* investigation also exposed that several of the experts present at this meeting had earlier been participating in Roche-sponsored events.^54^ Such episodes show that one of the ways that commercial backers of innovative molecular therapies may seek to maximise the return on their investment is by seeking to influence governments to procure sizeable quantities of their new products in the name of strengthening health security.^55^ The ways in which new molecular knowledges attract and coalesce with – politically influential – biocapital is thus another crucial driver behind the pharmaceutical turn in security policy.

## V. Therapeutic citizens: Changing patient behaviour and the Internet

Once new molecular therapies are commercially developed, governments can also come under intense political pressures from ‘below’ to procure the latest medical countermeasures on behalf of their citizens. The social anthropologist Vinh-Kim Nguyen has worked with the concept of ‘therapeutic citizenship’ to capture the multiple ways in which biomedical knowledge is shaping the government of both human (and nonhuman) affairs.^56^ He has traced how such knowledge is giving rise to complex new forms of association, activism, and exchange, which he was witnessing around access to antiretroviral therapy for people living with HIV/AIDS. Although Nguyen's notion of therapeutic citizenship is wide-ranging and complex, it is also underpinned by a strong expectation by citizens and patients that they should have rapid and affordable access to the latest pharmaceutical treatments.

Even beyond the contested politics of HIV/AIDS,^57^ there are signs that citizens are becoming much more proactive in seeking out the latest pharmaceutical regimens – often facilitated by recourse to the Internet.^58^ In the case of *Tamiflu*, direct-to-consumer advertising of the drug was not permitted in Europe due to legal constraints, but extensive media coverage of the pandemic threat certainly made citizens aware of the existence of this new drug. Bolstered by this awareness, citizens could actively seek out the information about this therapy on their own accord. The extent to which citizens were trying to obtain information about *Tamiflu*, and perhaps even trying to acquire the drug over the Internet, can be gleaned through looking at Google Trends data. Google Trends analyses a sample of searches performed on the commercial search engine, and then computes how many searches are being performed for a particular term relative to the number of searches done over time. These results are displayed in the Search Volume Index. Although this only provides a rough approximation due to the use of data sampling methods and multiple approximations, it clearly shows the enormously increased Internet activity surrounding *Tamiflu* during recent pandemic scares. In the graph below, the number 100 represents the peak search interest.

**Graph 1.**
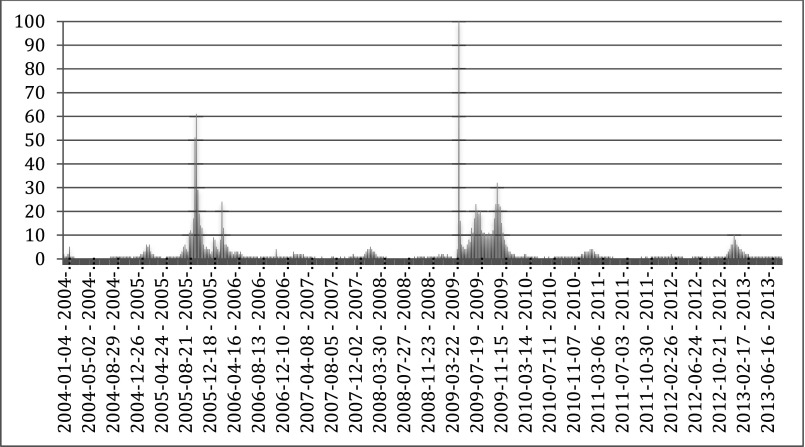
Internet activity surrounding *Tamiflu* during recent pandemic scares

*Source*: Google Trends 2013.

The Search Volume Index for ‘*Tamiflu*’ indicates two distinct spikes: one during the international fears of an imminent H5N1 pandemic in 2005, and one during the H1N1 pandemic that begun to spread in the April of 2009. Those peaks coincide with periods of intense media reporting.^59^

Such data suggest that many citizens desire information about, and actively seek access to, pharmaceutical treatments – especially during an emergency. There is also further evidence that if governments do not proactively provide access to such pharmaceutical therapies, citizens will go to considerable lengths to access them by other means. During the H5N1 ‘bird’ flu pandemic scare, for example, the poplar internet auction site ‘EBay’ had to withdraw sales of *Tamiflu* through its website after prices reached more than £100 for a treatment course – more than three times its usual prescription price.^60^ During the subsequent H1N1 ‘swine’ flu pandemic in April 2009, and despite UK government reassurances that the national stockpile was sufficiently large, online pharmacists again reported very dramatic increases in demand for *Tamiflu* as people tried to create personal stockpiles, in some cases demand was reportedly up by around 1,000 per cent.^61^ Faced with imminent health security threats, many citizens will actively seek information about, and demand access to, available pharmaceutical defences.

Those citizen expectations that they should enjoy access to the latest treatments also create tacit political pressure for governments – especially popularly elected ones – to make such pharmaceuticals more widely available. Explaining some of the thinking behind the UK decision to create an antiviral stockpile, Professor Robert Dingwall, who was a member of the UK Committee on Ethical Aspects of Pandemic Influenza, explained that: ‘It was felt it'd simply be unacceptable to the UK population to tell them we had a huge stockpile of drugs but they were not going to be made available.’^62^ The decision also reportedly came against a background a feelings amongst officials that ‘a fear of inaction and unpreparedness by the Department of Health which would lead to cataclysmic disapproval in the media’.^63^ The ways in which notions of citizenship are evolving around access to pharmaceutical treatments, in conjunction with popular expectations about state provision of pharmaceutical defences in the event of an emergency, are thus further drivers of the pharmaceutical turn in the security policy. When it comes to medical countermeasures, governments can find themselves being effectively ‘squeezed’ from both sides – not just by commercial pressures, but also by rising popular expectations to ensure widespread access to the latest pharmaceutical treatments.

## VI. Flexible pharmaceutical regulation: Governments and the bioeconomy

Caught in the crosshairs of those combined commercial and political pressures, governments are also adapting their regulatory regimes to make innovative pharmaceuticals more readily accessible. Those processes of regulatory adaptation mark a final piece of the pharmaceuticalisation ‘puzzle’ because pharmaceutical products are historically some of the most highly regulated products in the world. In most high-income countries any new pharmaceutical product needs to obtain official regulatory approval before it can be prescribed. This is true even if the government administers it. The extensive pharmaceutical turn in security policy witnessed over the past decade could not, therefore, have unfolded without a significant degree of regulatory adaptation by governments.

Again the case of *Tamiflu* is instructive. In the United States, the process of obtaining regulatory approval for *Tamiflu* coincided with a period of profound change in the regulatory approach taken by the Food and Drug Administration (FDA). In the late 1990s, and in a social context where many people were dying from AIDS-related illnesses, both patient groups and companies pressured the FDA to speed up and ease approval processes for new antiviral therapies. *Tamiflu* was not the first neuraminidase inhibitor to come up before the FDA for approval. That distinction went to its closely related predecessor drug *Relenza*, which FDA handled through a new priority review process lasting only six months.

During this *Relenza* approval process the Harvard-trained FDA statistician – Mike Elashoff – raised significant concerns about whether the efficacy of the drug had been established by the clinical trial data. Weighing up the evidence and discussion, the advisory committee overwhelmingly sided with the FDA statistician, and decided *not* to recommend approval of the drug by a significant majority of 13 votes to 4.^64^ That indicated a low chance of approval for the new drug, much to the dismay of its commercial developer. Months later, however, senior management at FDA nevertheless granted *Relenza* marketing approval, in part citing concerns about a possible future pandemic. In an extensive interview conducted for this research project, the same statistician – who had initially also been tasked with statistically evaluating the data on the other neuraminidase inhibitor *Tamiflu* – revealed that he was subsequently removed from the *Tamiflu* brief, which was then passed on to another statistician.^65^ When *Tamiflu* come up for regulatory approval a few months later, it was then approved by the FDA *without* recourse to an advisory committee – in part justified on the basis that the precedent for a neuraminidase inhibitor had already been established by the approval of *Relenza.*^66^

In the years that followed, governments also went on to systematically introduce an array of more flexible regulatory processes specifically governing the approval of medical countermeasures.^67^ Those novel pathways mean that, in the United States, some medical countermeasures can now be approved on the basis of animal efficacy studies rather than human clinical trials – thus easing the threshold for regulatory approval. New procedures were also introduced authorising the US government – in an emergency situation – to use a drug that had not yet secured regulatory approval, or the use of the drug for purposes other than those for which it was initially licenced.

In Europe, furthermore, the European Medicines Agency initiated three separate procedures for speeding up the availability of influenza vaccines during a pandemic. Those include: (1) a ‘mock-up procedure’ whereby a vaccine can be authorised in advance of a pandemic on the basis of a strain that could potentially cause a pandemic; (2) an ‘emergency procedure’, which reduces the authorisation procedure from 210 to seventy days; and (3) a ‘modification’ procedure whereby a ‘seasonal’ flu vaccine might be altered to afford protection against a pandemic strain.^68^ Without such enhanced regulatory flexibility, it would be more difficult for many medical countermeasures to gain the necessary regulatory approval, and companies would face greater disincentives for investing in the costly development of novel medical countermeasures. The willingness of governments to make their regulatory approaches for approving medical countermeasures more flexible is thus a crucial, final factor in ‘unlocking’ the pharmaceutical turn in security policy.

## Conclusion

Pharmaceuticals have become a much more prominent feature in the twenty-first-century landscape of security policy. Governments with the requisite resources now actively incentivise the commercial development of new medical countermeasures – through the design of novel programmes, through the use of public funds, through the creation of new institutions, and through the introduction of greater regulatory flexibilities. Governments are also building extensive pharmaceutical stockpiles that require continuous maintenance and replenishment, and are even standing up elaborate new logistical systems for distributing those medical countermeasures *en masse* to their populations outside of clinical settings. Indicators abound, then, that pharmaceuticals are becoming more vital to the task of securing populations.

At the core of that pharmaceutical turn in security policy lies the rise of a molecular vision of life promulgated by the biomedical sciences. Reimagining life as the complex interplay of molecular processes is provoking profound new fears about our collective vulnerability to underlying microbiological processes – be it in the form of pandemics or bioterrorism – that are finding their contemporary political expression in the rise of global health security concerns. At the same time, and as the detailed examination of *Tamiflu* showed, this molecular vision of life is simultaneously engendering new strategies for intervening upon life processes – principally by enabling the scientific development of an array of new medical countermeasures designed at the level of the molecular. How we understand life does not just shape what makes us feel insecure, but also influences how we in turn seek to secure life.

That it should be possible for security policy to undergo such a process of pharmaceuticalisation reaffirms the essentially malleable and socially constructed view of security as a complex field with boundaries that are subject to constant social renegotiation and contestation.^69^ That can be seen most evidently in the way that health security considerations have already begun to expand the borders of the security concept by incorporating many pressing health-based threats. It can further be seen in the increased role that medical experts now play in the formulation and implementation of security policy. And it can be seen by the increased recourse to pharmaceutically-based security strategies.^70^ Here the rise of global health security and its attendant quest for medical countermeasures forms part of a complex political negotiation – sometimes explicit, yet frequently implicit – of where the boundaries of security policy lie in an age of emergent molecular vulnerabilities.

But the pharmaceuticalisation perspective also contributes more to security studies than this core insight alone. Pharmaceuticalisation theory further captures the subtle ways in which our contemporary understandings, practices, and boundaries of security policy are – to no small measure – shaped by the broader social forces of health and biomedicine. Put differently, approaching global health security from the perspective of pharmaceuticals reveals that how we understand and practice security today cannot be divorced form our underlying and evolving conceptions of life, health and medicine.

That ultimately also tells us something deeper about the contours of contemporary world politics. At a level of considerable simplification and generalisation, we could say that – as a discipline – International Relations has long sought to explain the workings of international politics through recourse to the conceptual frameworks of politics, economics, or law – whether it is the structuring role of anarchy, of capitalism, of norms, or indeed the interaction of them all. Although that can hardly be considered an exhaustive list, one is certainly hard pressed to find scholarly accounts of international politics that afford health and medicine a prominent – not to mention constitutive – role. Even where the importance of health issues has occasionally been acknowledged in the scholarship of international politics, the discipline has tended to view them as second-order matters of ‘low’ politics when compared to the ‘high’ politics of international security.

The analysis of the pharmaceuticalisation of security presented here suggests something quite different. It shows that the social forces of health and biomedicine are much more formidable than this received disciplinary picture would suggest. That can be seen in terms of how infectious disease threats – from HIV/AIDS and SARS through to bird (H5N1) flu, swine flu (H1N1), and Ebola – now routinely attract the highest levels of international diplomatic efforts. It can also be seen in the way that international institutions – like the World Health Organization – are emerging as conduits of these social forces by pushing for wider access to pharmaceutical treatments, by recommending pharmaceutical strategies to member states, and by coordinating the international creation of new pharmaceutical stockpiles. And it can be seen in the ways that biomedicine is beginning to influence how states understand security, how they practice it, and indeed what it means for citizens to feel insecure. Far from merely being matters of ‘low’ politics, the social forces of health and biomedicine are powerful enough to influence the core practices of international politics – even those of security. For a discipline long accustomed to studying macrolevel processes and systemic structures, international relations are – in the end – also engendered and constituted by our knowledge of the minute morass of molecules.

## Footnotes

1 World Health Assembly (WHA). ‘Global health Security – Epidemic Alert and Response’, Report by the Secretariat prepared for the Fifty-Fourth World Health Assembly, A54/9 (Geneva: World Health Assembly, 2001); Global Health Security Initiative (GHSI), ‘Ministerial Statement’, London (March 2002), available at: {http://ghsi.ca/english/statementlondonmar2002.asp} accessed 8 April 2014; World Health Organization (WHO), ‘A Safer Future: Global Public Health Security in the 21st Century’, The World Health Report (Geneva: World Health Organization, 2007); European Council, ‘Council Conclusions on Health Security’ (Brussels: Council of the European Union, 16 December 2008); European Commission, ‘Commission Staff Working Document on Health Security in the European Union and Internationally’ (Brussels: Commission of the European Communities, 23 November 2009); StefanElbe, Virus Alert: Security, Governmentality and the AIDS Pandemic (New York: Columbia University Press, 2009); StefanElbe, Security and Global Health: Towards the Medicalization of Insecurity (Cambridge: Polity, 2010).

2 WHO, ‘A Safer Future’, p. ix.

3 Ibid., p. 47.

4 White House, ‘The National Security Strategy of the United States of America’ (Washington, DC: September 2002), p. vi.

5 White House, ‘The National Security Strategy of the United States of America’ (Washington, DC: March 2006), p. 47.

6 Cabinet Office, ‘The National Security Strategy of the United Kingdom: Security in an Interdependent World’ (London: The Cabinet Office, 2008), p. 3.

7 Cabinet Office, ‘A Strong Britain in an Age of Uncertainty: The National Security Strategy’ (London: The Cabinet Office, 2010), p. 27.

8 Irish Presidency of the European Union, ‘Presidency Confirms Agreement on Strengthening Responses to Serious Cross-Border Health Threats’, available at: {http://www.eu2013.ie/news/news-items/20130516crossborderhealthupdateengonly/} accessed 28 May 2013.

9 AdamKamradt-Scott and ColinMcInnes, ‘The securitisation of pandemic influenza: Framing, security and public policy’, Global Public Health, 7: Supp 2: S95–S110 (2012)2303905410.1080/17441692.2012.725752; see also Elbe, *Virus Alert*; and Elbe, *Security and Global Health*.

10 StefanElbe, ‘Should HIV/AIDS be securitized?’, International Studies Quarterly, 50:1 (2006), pp. 119–44; SaraDavies, ‘Securitizing infectious disease’, International Affairs, 84:2 (2008), pp. 295–313; ColinMcInnes and SimonRushton, ‘HIV/AIDS and securitization theory’, European Journal of International Relations, 19:1 (2013), pp. 115–38.

11 Elbe, *Security and Global Health*.

12 SimonRushton and Simon and JeremyYoude (eds), Routledge Handbook of Global Health Security (Oxford: Routledge, 2014); see also Joao Nunes, ‘Questioning health security: Insecurity and domination in world politics’, this Special Issue.

13 See also Alison Howell, ‘The global politics of medicine: Beyond global health, against securitization theory’, this Special Issue.

14 Institute of Medicine (IOM), ‘Medical Countermeasure Dispensing: Emergency Use Authorization and the Postal Model’, Workshop Summary (Washington, DC: The National Academies Press, 2010), p. 5.

15 US Department of Health and Human Services (HHS), ‘2012 HHS PHEMCE Strategy and Implementation Plan’ (Washington, DC, 2012), available at: {http://www.phe.gov/Preparedness/mcm/phemce/Pages/strategy.aspx} accessed 13 March 2013.

16 IOM, ‘Medical Countermeasure Dispensing’, p. 6.

17 Biomedical Advanced Research and Development Authority (BARDA), ‘BARDA Strategic Plan: 2011–2016’ (Washington, DC, 2011), p. 1, available at: {http://www.phe.gov/about/barda/Pages/2011barda-stratplan.aspx} accessed 8 April 2014.

18 Ibid.

19 US Department of Health and Human Services (HHS), ‘Project BioShield Annual Report to Congress January 2011 – December 2011’ (Washington, DC: Office of the Assistant Secretary for Preparedness and Response, 2012).

20 Chris Schneidmiller, ‘Q&A: bioshield program successful after “Rocky Start”, HHS Preparedness Chief says’, *Global Security Newswire* (1 March 2013), available at: {http://www.nti.org/gsn/article/q-bioshield-program-successful-after-rocky-start-hhs-preparedness-chief-says/} accessed 28 June 2013.

21 HHS, ‘Project BioShield Annual Report’.

22 Tara KirkSell and MatthewWatson, ‘Federal agency biodefense funding, FY2013–FY2014’, Biosecurity and Bioterrorism, 11:3 (9 2013), pp. 196–2162390600910.1089/bsp.2013.0047PMC3778993.

23 Stephen Prior, ‘Who You Gonna Call? Responding to a Medical Emergency with the Strategic National Stockpile’, Report Commissioned by the National Defense University, Center for Technology and National Security Policy (Arlington, VA: Potomac Institute for Policy Studies, June 2004).

24 IOM, ‘Medical Countermeasure Dispensing’, p. 6.

25 Stephen Prior, ‘Who You Gonna Call?’, p. 7.

26 Todd Piester, ‘Strategic National Stockpile: Pandemic Influenza Countermeasures’, Presentation: Centers for Disease Control and Prevention (Atlanta, GA: 21 August 2008).

27 Stephen Prior, ‘Who You Gonna Call?’, p. 2.

28 Irish Presidency of the EU, ‘Presidency Confirms Agreement on Strengthening Responses’.

29 Department of Health, ‘National Medical Stockpile’, Australian Government Department of Health, available at: {http://www.health.gov.au/internet/main/publishing.nsf/Content/health-pubhlth-strateg-bio-factsht_stckpile.htm} accessed 5 March 2014.

30 Public Health Agency of Canada (PHAC), ‘National Emergency Stockpile System’ (2012), available at: {http://www.phac-aspc.gc.ca/ep-mu/ness-eng.php} accessed 5 March 2014.

31 UK Department of Health, ‘Emergency Planning: UK Reserve National Stock for Major Incidents’ (London: United Kingdom Department of Health, 2007).

32 SandraMounier-Jack and RichardCoker, ‘How prepared is Europe for pandemic influenza? Analysis of national plans’, The Lancet, 367 (2006), p. 140810.1016/S0140-6736(06)68511-516650650.

33 Roche Pharmaceuticals, ‘Preparing for and Responding to Influenza Pandemics: Roles and Responsibilities of Roche’ (June 2012), available at: {www.roche.com/roles_responsibilities_influenza.pdf} accessed 25 February 2013.

34 RaminaSamii and Lukvan Wassenhove, Fighting the Flu: Tamiflu Stockpiling: A Pandemic Preparedness Policy (Fontainebleau: Insead, 2008), p. 5.

35 NeilFerguson, D.Cummings, S.Cauchemez, C.Fraser, S.Riley, A.Meeyai, S.Iamsirithaworn, and D.Burke, ‘Strategies for containing an emerging influenza pandemic in Southeast Asia’, Nature, 437:7056 (2005), p. 2091607979710.1038/nature04017.

36 Samii and Wassenhove, *Fighting the Flu*, p. 5.

37 Ibid.

38 Maureen Baker, ‘Quality Assuring the NPFS … and Further RCGP Reflections on Pandemic H1N1 (2009)’, A Report on the NPFS and the H1N1 Swine Flu Outbreak for the Chief Medical Officer (Personal Correspondence, 2010), p. 7.

39 Sandra Peek, ‘The distribution of Tamiflu is open to widespread abuse’, letter to *The Telegraph* (29 July 2009), available at: {http://www.telegraph.co.uk/comment/letters/5926603/The-distribution-of-Tamiflu-is-open-to-widespread-abuse.html} accessed 8 July 2010.

40 Maureen Baker, ‘Quality Assuring the NPFS’.

41 AndrewLakoff, Pharmaceutical Reason: Knowledge and Value in Global Psychiatry (Cambridge: Cambridge University Press, 2005); JohnAbraham, ‘Pharmaceuticalization of society in context: Theoretical, empirical and health dimensions’, Sociology, 44:4 (2010), pp. 603–22; S.Williams, P.Martin, and J.Gabe, ‘The pharmaceuticalisation of society? An analytical framework’, Sociology of Health and Illness, 33:5 (2011), pp. 710–252137104810.1111/j.1467-9566.2011.01320.x.

42 NikolasRose, The Politics of Life Itself: Biomedicine, Power, and Subjectivity in the Twenty-First Century (Princeton, NJ: Princeton University Press, 2007), pp. 5–6; see also Lily E.Kay, The Molecular Vision of Life: Caltech, the Rockefeller Foundation, and the Rise of the New Biology (New York: Oxford University Press, 1993); Lily E.Kay, Who Wrote the Book of Life?: A History of the Genetic Code (Stanford: Stanford University Press, 2000)10.1023/a:101033281101714513856; MichaelDillon and JulianReid, ‘Global liberal governance: Biopolitics, security and war’, Millennium, 30:1 (2001), pp. 41–66.

43 BruceBraun, ‘Biopolitics and the molecularization of life’, Cultural Geographies, 14:1 (2007), pp. 6–28.

44 AngusNicoll and MarcSprenger, ‘Learning lessons from the 2009 pandemic: Putting infections in their proper place’, European Journal of Epidemiology, 26:3 (2011), pp. 191–4, 1912148795710.1007/s10654-011-9575-4PMC3079088.

45 DavidReddy, ‘Responding to pandemic (H1N1) 2009 influenza: The role of oseltamivir’, Journal of Antimicrobial Chemotheray, 65: Suppl 2:ii (2007), pp. 35–4010.1093/jac/dkq014PMC283551020215134.

46 Roche, ‘Media Release’ (26 April 2007), available at: {http://www.roche.com/media/media_releases/med-cor-2007-04-26.htm} accessed 3 September 2013, p. 12.

47 Reto Schneider, ‘Das Rennen um GS4104: Wie ein Medikament entwickelt, getestet und vermarktet wird’, *NZZ Folio: Die Zeitschrift der Neuen Zuercher Zeitung* (April 2001), available at: {http://www.nzzfolio.ch/www/d80bd71b-b264-4db4-afd0-277884b93470/showarticle/81bb3c96-9216-4eb5-b602-7e0937369c79.aspx} accessed 1 March 2012.

48 KaushikRajan, Biocapital: The Constitution of Postgenomic Life (Durham, NC: Duke University Press, 2006), p. 78; see also MelindaCooper, Life as Surplus: Biotechnology and Capitalism in the Neoliberal Era (Seattle, WA: Washington University Press, 2008).

49 TamasBartfai and GrahamLees, The Future of Drug Discovery: Who Decides Which Diseases to Treat (London: Elsevier, 2013), p. 275.

50 DeborahCohen and PhilipCarter, ‘Conflicts of interest: WHO and the pandemic flu “conspiracies”’, BMJ, 340 (2010), pp. 1274–910.1136/bmj.c291220525679.

51 World Health Organization, ‘Influenza Pandemic Preparedness Plan. The Role of WHO and Guidelines for National or Regional Planning’ (Geneva: World Health Organization, April 1999), p. 54.

52 Ibid., p. 54.

53 Cohen and Carter, ‘Conflicts of interest’, p. 1275.

54 Ibid.

55 On the role of pharmaceutical companies in global health governance more generally, see Anne Roemer-Mahler, ‘The rise of companies from emerging markets in global health governance: Opportunities and challenges’, this Special Issue.

56 Vinh-KimNguyen, The Republic of Therapy: Triage and Sovereignty in West Africa's Time of AIDS (Durham, NC: Duke University Press, 2010); see also AdrianaPetryna, Biological Citizenship: The Science and Politics of Chernobyl-Exposed Populations (Princeton, NJ: Princeton University Press, 2013)10.1086/64940515484388.

57 See Garrett W. Brown, ‘Norm diffusion and health system strengthening: The persistent relevance of national leadership in global health governance’, this Special Issue.

58 PeterConad, The Medicalization of Society: On the Transformation of Human Conditions into Treatable Disorders (Baltimore: Johns Hopkins University Press, 2007); Nguyen, *The Republic of Therapy.*

59 Google Trends. Search performed on ‘Tamiflu’, available at: {http://www.google.co.uk/trends/explore?q=Tamiflu#q=Tamiflu&cmpt=q} accessed 2 September 2013.

60 Reuters, ‘EBay stops Tamiflu sale on Web as drug price soars’, Reuters (18 October 2005), available at: {http://www.redorbit.com/news/health/275417/ebay_stops_tamiflu_sale_on_web_as_drug_price_soars/index.html} accessed 13 July 2010.

61 Jon Swaine and Rebecca Smith, ‘Swine flu: Online pharmacies report huge surge in demand for Tamiflu’, *The Telegraph* (27 April 2009), available at: {http://www.telegraph.co.uk/health/swine-flu/5231337/Swine-flu-Online-pharmacies-report-huge-surge-in-demand-for-Tamiflu.html} accessed 8 July 2010.

62 Simon Garfield, ‘Catch it! Bin it! Profit from it!’, *The Observer* (25 October 2009), available at: {http://www.guardian.co.uk/world/2009/oct/25/swine-flu-vaccines-profit/print} accessed 2 September 2013.

63 Ibid.

64 FDA, ‘Food and Drug Administration Centre for Drug Evaluation and Research: Antiviral Drugs Advisory Committee’ (Gaithersburg, MA: 24 February 1999).

65 Mike Elashoff, Interview, San Francisco, 31 March 2012.

66 Ibid.

67 StefanElbe, ‘Medical countermeasures for national security: A new government role in the pharmaceuticalization of society’, Social Science & Medicine (forthcoming, 2014)10.1016/j.socscimed.2014.04.03524815579.

68 European Medicines Agency (EMA), ‘Authorization Procedures’ (London: European Medicines Agency, 2014), available at: {http://www.ema.europa.eu/ema/index.jsp?curl=pages/special_topics/q_and_a/q_and_a_detail_000080.jsp} accessed 6 March 2014.

69 MichelFoucault, Security, Territory, Population: Lectures at the Collège de France 1977–1978, trans. GrahamBurchell (New York: Palgrave, 2007); PierreBourdieu, ‘The social space and the genesis of groups’, Theory and Society, 14:6 (1985), p. 734; DidierBigo, ‘Pierre Bourdieu and International Relations: Power of practices, practices of power’, International Political Sociology, 5 (2011), pp. 225–8.

70 Stefan Elbe, *Security and Global Health.*

